# Comparative Transcriptome Analysis Reveals the Potential Mechanism of Abortion in Tobacco *sua*-Cytoplasmic Male Sterility

**DOI:** 10.3390/ijms21072445

**Published:** 2020-04-01

**Authors:** Zhiwen Liu, Yanfang Liu, Yuhe Sun, Aiguo Yang, Fengxia Li

**Affiliations:** 1 Tobacco Research Institute, Chinese Academy of Agricultural Sciences, Qingdao 266101, China; 2 Department of Key Laboratory for Tobacco Gene Resources, State Tobacco Monopoly Administration, Qingdao 266101, China; 3 Graduate School of Chinese Academy of Agricultural Science, Beijing 100081, China

**Keywords:** tobacco, transcriptome, programmed cell death, cytoplasmic male sterility, ATP synthesis

## Abstract

*sua*-CMS (cytoplasmic male sterility) is the only male sterile system in tobacco breeding, but the mechanism of abortion is unclear. Cytological characteristics show that abortion in the *sua*-CMS line msZY occurs before the differentiation of sporogenous cells. In this study, a comparative transcriptomic analysis was conducted on flower buds at the abortion stage of msZY and its male fertile control ZY. A total of 462 differentially expressed genes were identified in msZY and ZY, which were enriched via protein processing in the endoplasmic reticulum (ER), oxidative phosphorylation, photosynthesis, and circadian rhythm-plant by Kyoto Encyclopedia of Genes and Genomes (KEGG) pathway analyses. Most genes were downregulated in the ER stress pathway, heat-shock protein family, F1F0-ATPase encoding by the mitochondrial genome, and differentiation of stamens. Genes in the programmed cell death (PCD) pathway were upregulated in msZY. The transcriptome results were consistent with those of qRT-PCR. Ultrastructural and physiological analyses indicted active vacuole PCD and low ATP content in msZY young flower buds. We speculated that PCD and a deficiency in ATP synthesis are essential for the abortion of *sua*-CMS. This study reveals the potential mechanism of abortion of tobacco *sua*-CMS.

## 1. Introduction

Cytoplasmic male sterility (CMS) is a pollen abortion process controlled by the cytoplasmic genome. It is an important way of exploiting the heterosis of crops and has significantly enhanced agricultural productivity [1]. CMS genes are often mitochondrial encoded factors, which are generated by rearrangement and recombination of mitochondrial genomes [2]. Pollen development is a highly orchestrated event, and gene and transcription factors (TFs) controlling pollen development are located in the nuclear genome. CMS genes may regulate the expression of these nuclear genes, and the plant CMS system is thus an ideal model for studies on nucleo-cytoplasmic interactions [3,4].

Sanders et al. [5] artificially divided anther development of *Arabidopsis thaliana* into 14 stages. In stages 1−3, the stamen primordia differentiates into sporogenous cells. In stages 4−8, the differentiated microsporocytes develop into quarter microspores. In stages 9−14, the microspores develop into mature pollen grains. In most types of CMS, abortion mainly occurs during microsporogenesis (stages 4−8) [6,7] and pollen maturation (stages 9−14) [8,9,10,11]. The molecular mechanism of CMS in these stages has been studied in detail [12]. However, there are fewer abnormalities in the early stages of anther development (stages 1−3) [13,14,15], and the molecular mechanism of abortion in these stages is unclear.

Stamen organogenesis of angiosperms is mainly controlled by the overlapping activity of B genes *APETALA3*(*AP3*) and *PISTILLATA* (*PI*), the C gene *AGAMOUS* (*AG*), and E gene *SEPs* in the expanded ABC(DE) model [16]. *NOZZLE/SPOROCYTELESS* (*NZZ/SPL*) is directly downstream of *AG*, and is involved in the differentiation of sporogenous cells [17,18]. *AG* and *AP3*/*PI* respectively interact with sites in the 3′ and 5′ regions of *NZZ/SPL*, and activate the expression of NZZ/SPL [19]. *AP3* is downregulated in *Brassica campestris* CMS lines [20]. Genes involved in early anther differentiation, including *NZZ/SPL*, are downregulated in the Shaan2A CMS line of *B. napus* [21].

Plant traits, such as development, productivity, fertility, and resistance, need to consume chemical energy (ATP)—especially anther development, which is a highly energy-consuming process [9,22]. Energy is generated by oxidative phosphorylation (OXPHOS), glycolysis, and the tricarboxylic acid cycle (TCA cycle). The most productive is OXPHOS in mitochondria, in which a glucose molecule produces 26 ATP molecules, while only two ATP molecules are produced in both the glycolysis pathway and the TCA cycle. OXPHOS consists of NADH dehydrogenase (complex I), succinate dehydrogenase (complex II), ubiquinol-cytochrome c reductase (complex III), cytochrome c oxidase (complex IV), and F1F0-ATPase (complex V) embedded in the inner mitochondrial membrane. Mutations in the complex subunits or reduced activity of the complexes could cause an imbalance of energy metabolism, leading to male sterility [3].

In the Honglian (HL)-CMS of rice, the sterilizing factor ORFH79 inhibits the activity of complex III, reducing the ATP level and increasing reactive oxygen species (ROS) content [10]. Changes in complex V are more likely to reduce ATP production. Downregulation of the delta subunit of F1F0-ATPase reduces mitochondrial ATP level and restricts anther development in *Arabidopsis* [23]. Interaction of ORF507 with the ATP6 subunit reduces the activity of F1F0-ATPase in mitochondria and causes abortion in chili pepper [24]. ORF522, a gene that is correlated with PET1-CMS of the sunflower, competes with the subunit of F1F0-ATPase ORFB (atp8) and reduces F1F0-ATPase activity [25]. Over-expression of the unedited orfB in male fertile indica rice [26], or of atp9 in *Nicotiana tabacum* [27], results in low F1F0-ATPase activity and generates male sterility.

The endoplasmic reticulum (ER) is a crucial site for protein folding. About one-third of plant proteins are folded and assembled in the ER [28]. The ER quality control (ERQC) machinery supervises the protein assembly process by promoting protein folding and the degradation of misfolded proteins. When plants are under environmental stress or treated with an ER stress agent, ERQC machinery cannot meet the needs of protein folding and misfolded proteins accumulate, causing ER stress [29]. Unfolded protein response (UPR), a cytoprotective signaling pathway induced by ER stress, transmits information about the protein folding status in the ER to the nucleus and increases protein folding capacity by upregulating ER chaperone genes in ERQC, including binding protein (*BiP*), calnexin (*CNX*), calreticulin (*CRT*), and protein disulfide isomerase (*PDI*) [30].

BiP is the most abundant chaperone protein in the ER lumen. It prevents protein aggregation and assists in correctly folding proteins [31]. BiP also senses stress and then activates the ER stress sensor, inositol-requiring enzyme 1 (IRE1), during UPR [32]. IRE1 splices and produces an active *bZIP60* TF [33]. bZIP60 subsequently enters into the nucleus and regulates target genes, such as *BiP*, *CNX*, *CRT*, and *PDI*, restoring ER proteostasis to help the protein fold properly [34]. ER-assisted degradation (ERAD) is another way to relieve ER stress. In the ERAD pathway, misfolded proteins are discharged to the cytosol for proteasomal degradation [30]. First, misfolded proteins are recognized by AtOS9, and they then interact with HRD3A and bind to the HRD1 complex. The HRD1 complex acts as a substrate for ubiquitinated UBC32 and degrades misfolded proteins through 26S proteasome degradation [35].

If excessive or persistent accumulation of misfolded proteins occurs in the ER, autophagy and programmed cell death (PCD) will be triggered [36]. PCD is a genetically determined self-activated cell death process initiated during normal development of an organism or under the influence of certain environmental factors [37]. PCD is an ordered process of the selective removal of cells, and it is essential for the growth and development of multicellular organisms, as well as for proper environmental responses [38]. Aberrant PCD is also the abortion mechanism of much CMS, mainly manifested by premature and delayed PCD of the tapetum in microsporogenesis or uninucleate stages [22,39,40,41].

In soybean, key genes involved in ER stress-induced PCD have been identified. *GmERD15* is a TF that is first induced by ER stress and then binds N-rich protein (NRP) promoters and activates NRP (NRP-A, NRP-B) expression. NRPs cause the upregulation of NAC (NAM, ATAF1, CUC2, NAC) TFs *GmNAC81* and *GmNAC30* [42]. NACs interact with each other in a synergistic manner to directly activate vacuolar processing enzyme (*VPE*) gene expression [43]. VPE is a plant protease that participates in plant PCD by controlling tonoplast rupture [44]. The Bax inhibitor 1 gene (*BI-1*) is a suppressor of PCD. Overexpression of *BI-1* inhibits ER-induced death during PCD [45]. *BI-1* antisense promotes apoptosis in tumor cell lines [46].

Heat-shock proteins (HSPs) are required for normal protein folding. HSPs are major components of multiple stress responses that act as primary mitigators of cell stress and play essential roles in developmental processes, as well as different kinds of environmental stress conditions [47,48]. Hsp70s potently counteract PCD and play an anti-apoptotic role in many cells [49]. Overexpressing *Hsp70* in rice inhibits PCD induced by heat and H_2_O_2_ in rice protoplasts [50].

The vacuole is a key organelle of plant PCD. Unlike phagocytosis in animals, unwanted cells in plants are degraded by the release of hydrolytic enzymes and destruction of cellular components, which results in rupture of the vacuolar membrane and vacuolar collapse [51]. Morphological characteristics of plant PCD include mitochondrial swelling, nuclear envelope disassembly and rupture of the plasma membrane at early stages, shrinkage of the protoplast, and rupture of the tonoplast and vacuolar cell death at the last stage [52]. Tonoplast rupture leads to the release of degrading enzymes from the vacuole into the cytoplasm and degraded cellular components [38]. In plant PCD, the VPE localized in vacuoles acts as an initiator by promoting the hydrolysis of vacuole proteins and the rupture of the vacuole [53].

The *sua*-CMS of tobacco was developed in the 1950s by somatic fusion between *N. tabacum* and *N. suaveolens* and repeated backcrossing [54]. It was the only male sterile system in tobacco breeding, and the planting area of *sua*-CMS cultivars accounted for more than 80% of the total tobacco planting area in China in 2018. Anthers of *sua*-CMS lines are mainly stigma-like, occasionally petal-like, or completely degenerated, depending on the environment (mainly affected by temperature). Therefore, abortion of anthers occurs at the early stage of anther development. There are six unique ORFs on the mitochondrial genome of *sua*-CMS line, through mitochondrial whole-genome sequencing and comparative genomes, which are expressed highly in anthers and floral buds of *sua*-CMS line [55]. Genes involved in early anther development are obtained through comparative transcriptomic analyses, focusing on the early anther development of the CMS plant and its male fertile control. In this study, we determined the abortive period of *sua*-CMS lines using histological sections. Then, the transcriptomes from *sua*-CMS line msZhongyan100 (msZY) and its male control Zhongyan100 (ZY) were sequenced and compared. The results showed that differentially expressed genes were involved in protein processing in the ER, oxidative phosphorylation, photosynthesis, and circadian rhythm-plant. Additionally, genes involved in the differentiation of stamens were downregulated in msZY. Abnormal energy metabolism and PCD may underlie the early developmental differences of msZY and ZY anthers. These findings provide valuable information for a better understanding of *sua*-CMS of tobacco at the molecular level and the interaction between the nuclear and mitochondrial genomes.

## 2. Results

### 2.1. Cytological Characteristics of Early Anther Development of msZY

**Figure 1:**
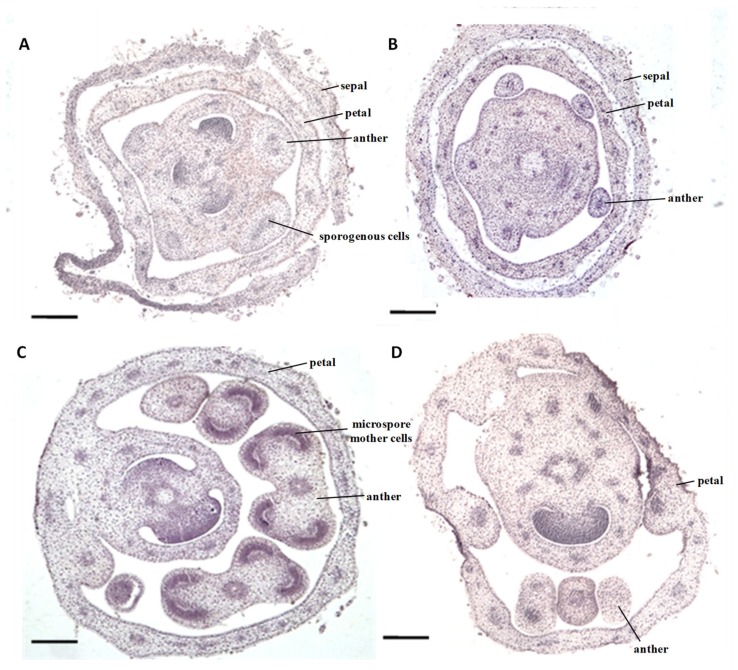
Microstructure of *sua*-CMS line msZY and its male fertile control ZY. (**A**,**B**) flower buds <2 mm: (**A**) ZY, (**B**) msZY. (**C**,**D**) flower buds of 2mm-3mm: (**C**) ZY, (**D**) msZY. Scale bar = 200 μm.

The size of the flower buds of the *sua*-CMS line msZY was basically the same as that of the male fertile control ZY, from young flower buds to the fully open flowers. Flower buds were, however, completely different in anther development. The flower buds 2 mm in size had differentiated sporogenous cells in anthers of ZY (Figure 1A). Flower buds of the same size had different numbers of anthers, but were not differentiated into sporogenous cells or even archesporial cells in the anthers of msZY; there was only a thick stained area in the central pith (Figure 1B). When the flower buds grew to 2-3 mm, four obvious anther locules and microspore mother cells were observed in the anthers of ZY (Figure 1C), while the anthers of msZY were not fully differentiated (Figure 1D). Abortion of msZY therefore occurred in the early stages of anther development, in which there were no sporogenous cells and the size of the flower buds did not exceed 2 mm. Therefore, flower buds of <2 mm were used for comparative transcriptome analysis in the present study.

### 2.2. RNA Sequencing and Identification of Differentially Expressed Genes (DEGs)

A comprehensive transcriptome of tobacco early anther development was obtained using the Illumina HiSeq 2000 by RNA sequencing. In this study, an average of 48.0 million raw reads per library were generated. After removing adaptor sequences, reads with >10% ambiguous nucleotides, and low-quality reads, an average of 46.2 million clean reads were obtained per library. The GC content and Q20 of the six samples were approximately 41.94% and 98.3%, respectively (Table S2). An average of 92.6% clean reads were mapped to *N. tabacum* reference sequences using HISAT2. The value of Pearson’s correlation coefficients for all biological replicates was >0.8, suggesting that the Illumina sequencing data were effective and could be used in further analyses.

To investigate the genes involved in early anther development, we identified putative differentially expressed genes (DEGs) between msZY and ZY using DESeq2 software. Padj (*p*-adjusted) ≤0.001 and |log2 foldchange| ≥1 were used as the thresholds to judge the significance of a difference in gene expression. A total of 462 DEGs were identified using this selection method. There were 225 upregulated and 237 downregulated genes in msZY compared to ZY (Figure S1).

### 2.3. Gene Ontology (GO) Enrichment and Kyoto Encyclopedia of Genes and Genomes (KEGG) Analysis of DEGs

To further understand which genes were related to *sua*-CMS of tobacco, gene ontology (GO) enrichment and KEGG analyses of DEGs were compared between msZY and ZY. The DEGs were enriched in 115 GO terms (*p*-value <0.05). The biological process category had 87 GO terms; the cellular components and molecular functions categories had 18 and 10 GO terms (*p*-value <0.05), respectively. The GO enrichment analysis showed that more DEGs were enriched in organonitrogen compound metabolic process (GO: 1901564), organophosphate biosynthetic process (GO: 0090407), membrane protein complex (GO: 0098796), monovalent inorganic cation transmembrane transporter activity (GO: 0015077), and hydrogen ion transmembrane transporter activity (GO: 0015078) (Supplementary Figure S2).

**Figure 2:**
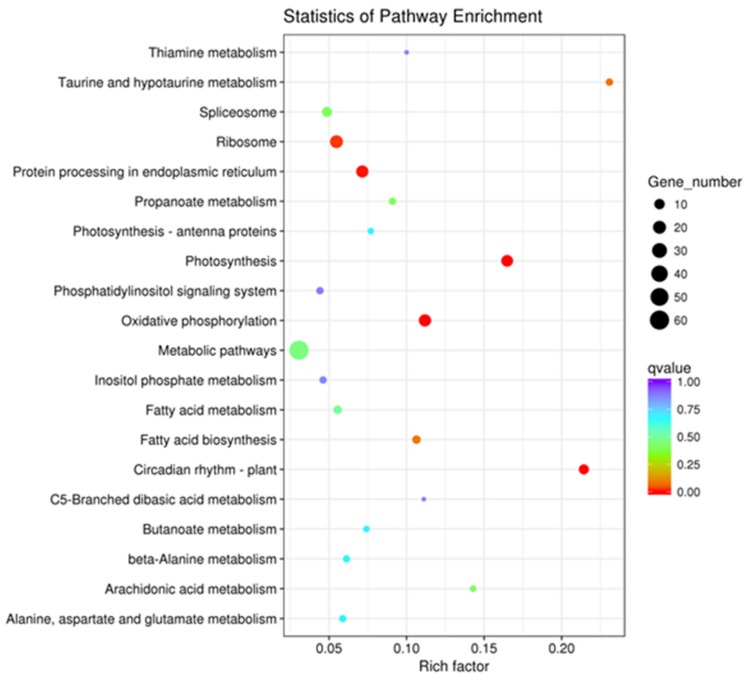
Scatter plot of Kyoto Encyclopedia of Genes and Genomes (KEGG) pathway enrichment statistics of differentially expressed genes (DEGs) between msZY and ZY.

The DEGs were enriched by KEGG functional annotations in 68 pathways, among which the significant (*p*-value <0.05) ones were protein processing in the endoplasmic reticulum (*n* = 17), oxidative phosphorylation (*n* = 17), photosynthesis (*n* = 15), circadian rhythm-plant (*n* = 9), and the ribosome (*n* = 20). The top 20 pathways enriched for up- and down-regulated DEGs are shownin Figure 2. These data provided an initial framework for screening DEGs involved in the*sua*-CMS of tobacco.

### 2.4. Verification of Differentially Expressed Genes by qRT-PCR

**Figure 3:**
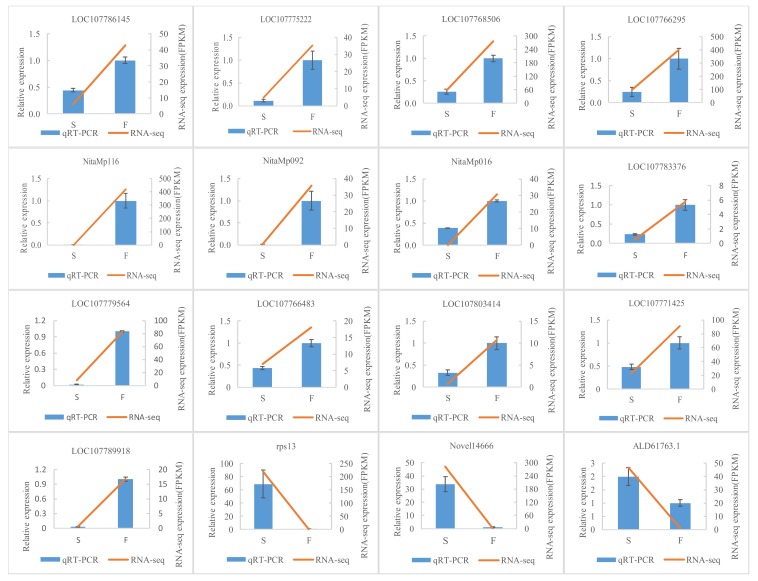
qRT-PCR verification diagram between flower buds of msZY and ZY. Comparison of the expression levels determined by qRT-PCR and RNA-seq from three biological replicates. Result were calculated using log2 fold variation measurements. All data indicate mean ± standard error (SE). LOC107786145, LHY-like(late elongated hypocotyl) protein; LOC107775222, LHY-like protein; LOC107768506, 70 kDa protein 2-likeheat shock cognate; LOC107766295, 70 kDa protein 2-likeheat shock cognate; NitaMp116, hypothetical protein, similar to orf124 in *Beta vulgaris*; NitaMp092 and orf306, hypothetical proteins; NitaMp016, hypothetical protein; LOC107783376, 70 kDa protein 2-likeheat shock cognate; LOC107766483, 70 kDa protein 15-likeheat shock; LOC107803414, 70 kDa protein 2-likeheat shock cognate; LOC107771425, binding protein (BiP); LOC107789918, SPOROCYTELESS (SPL)-like; rps13, ribosomal protein S13; Novel14666, photosystem II CP43 protein-like reaction center; and ALD61763.1, nad2 NADH dehydrogenase subunit 2.

Sixteen DEGs were detected by qRT-PCR to validate the transcriptome data. The selected genes were mainly in ER stress (LOC10777142), anther development (LOC107789918), HSP families (LOC107768506, LOC107783376, LOC107766483, LOC107803414, LOC107766295), energy metabolism (ALD61763.1, NitaMp092), cellular process (LOC107775222, rps13, LOC107779564), circadian rhythm (LOC107786145), and other unknown pathways (NitaMp116, NitaMp016, Novel14666). The results of qRT-PCR were consistent with the transcriptome data (Figure 3).

### 2.5. Genes Related to Stamen Development

Some genes related to the differentiation of stamens were identified. We analyzed the expression profiles of these genes in msZY and ZY. The expression level of *SPL/NZZ* (LOC107789918) in msZY was only 0.04 fold of that in ZY. B gene *PI* (LOC107803851) and C gene *AG* (LOC107761813) were also downregulated in msZY (Table 1).

### 2.6. Differentially Expressed Genes Related to Energy Metabolism

OXPHOS is a direct process forgenerating energy. F1F0-ATPase, the final component of OXPHOS, plays the central role in ATP production [56]. F1F0-ATPase is composed by the F1 part, the F0 part, and the peripheral stator, which includes 14 subunits in *A. thaliana* [57]. Transcriptome data analysis revealed that the encoding genes of F0–atp9, atp8, atp6, and stator–atp4 were downgraded 30%–40% in msZY compared to ZY (Table 1).

**Figure 4:**
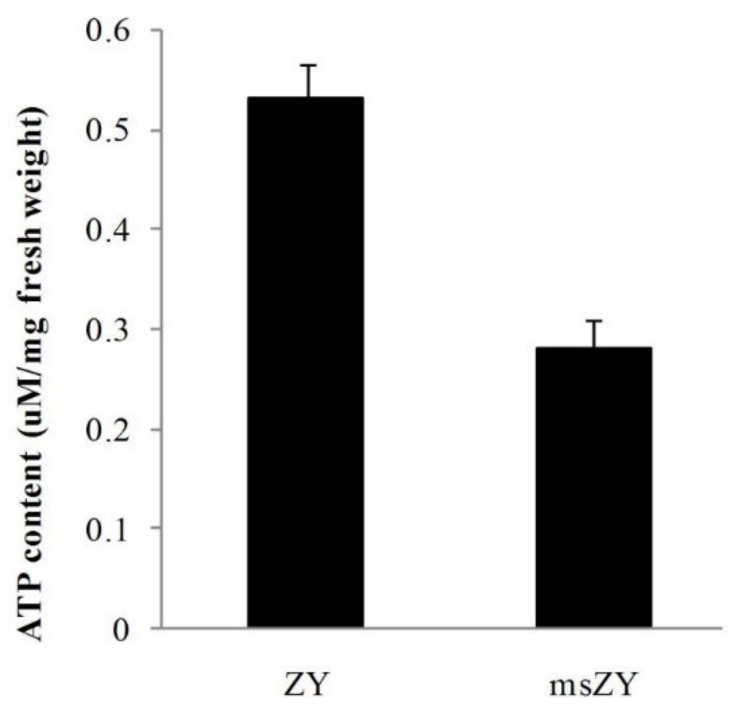
ATP content of flower buds of msZY and ZY.

We measured the ATP content of young flower buds of msZY and ZY. The results showed that the ATP content was 0.53 μM/mg fresh weight in ZY, while the ATP content of msZY was only 52% of ZY (Figure 4), indicating a deficiency of ATP synthesis in the early development of flower buds of msZY.

### 2.7. Differentially Expressed Genes in Endoplasmic Reticulum Stress and Programmed Cell Death

**Figure 5:**
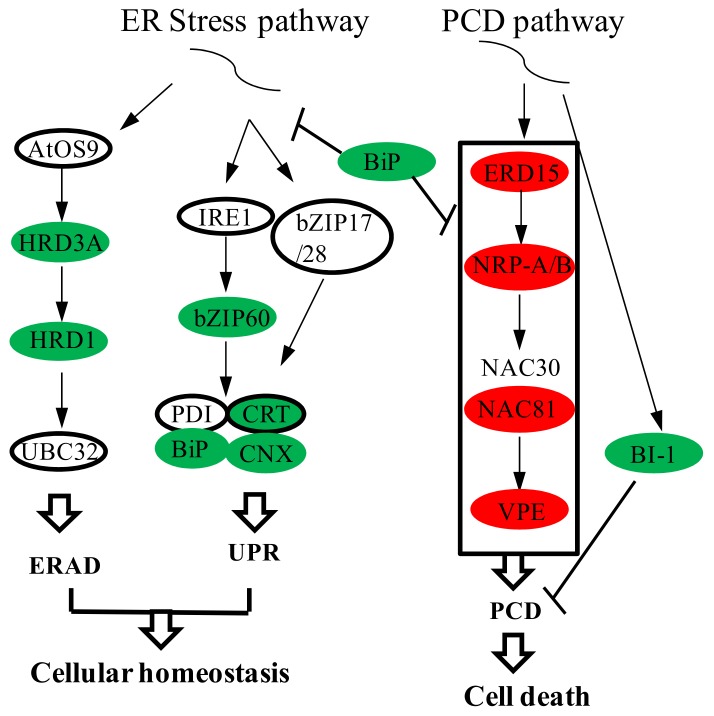
Gene expression in ER stress and PCD pathway. ER: endoplasmic reticulum; ERAD: ER-assisted degradation; UPR: Unfolded protein response; PCD: programmed cell death; BiP: binding protein; IRE1: inositol-requiring enzyme 1; PDI: protein disulfide isomerase; CRT: calreticulin; CNX: calnexin; VPE: vacuolar processing enzyme; BI-1:Bax inhibitor 1 gene; NRP: N-rich protein. Red indicates upregulated expression, and green indicates downregulated expression.

When organisms are subjected to certain endogenous or exogenous stresses, the probability of protein misfolding in the ER increases, causing ER stress. UPR and ERAD sense this stress, and respond to achieve new ER homeostasis by enhancing protein folding capacity and degrading misfolded proteins, respectively. Our results revealed that the DEGs enriched in protein processing in the ER were related to ER stress, and were downregulated in msZY (Figure 5). The expression level of *bZIP60* in young flower buds of msZY was 30% of that of ZY. BiP is a chaperone in the ER, and its expression level in msZY was only 40% of that of ZY. qPCR also confirmed that the expression level of *BiP* (LOC107771425) in young flower buds of msZY was much lower than that of ZY (Figure 3). The other two chaperone genes (*CNX* and *CRT*) in the ERQC system were also downregulated in msZY (Table 1). *HRD1* and *HRD3A*, which were the key genes in the ERAD pathway, were found to be downregulated in msZY (Table 1). We hypothesized that the low expression of genes related to ER stress in msZY young flower buds resulted in ER stress.

Sustained ER stress triggers ER stress-induced PCD. In this study, several genes identified in the plant PCD pathway—for example, *ERD15* (LOC107831960), *NRP-A/B* (LOC107799405, LOC107786583), *NAC81* (LOC107784516), and *VPE* (LOC107807349)—were upregulated in the flower buds of msZY (Figure 5). The expression level of *ERD15* in msZY was 3.7-fold higher than in ZY, and the expression level of NRP-A/B was also approximately 1.3-fold higher than in ZY. The cell death inhibitory factor BI-1 negatively regulates PCD. *BI-1* (LOC107803860) was downregulated in msZY (Table 1).

**Figure 6:**
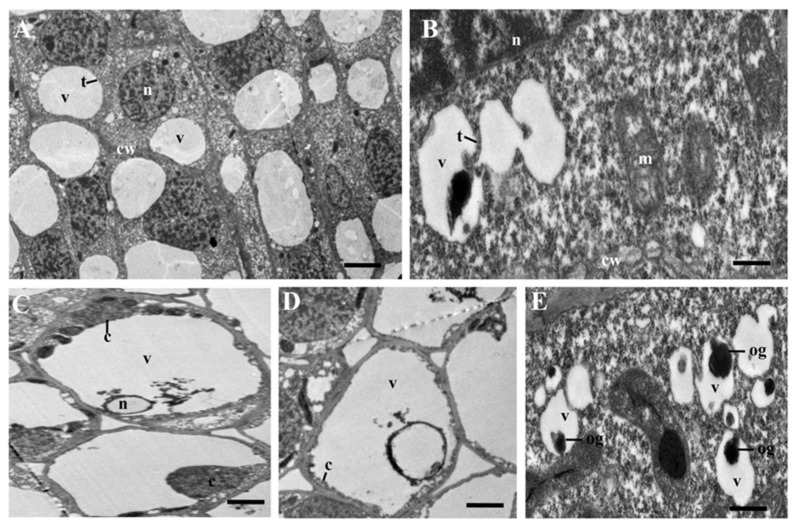
Transmission electron microscopy (TEM) micrographs of tobacco flower buds. (**A**) ZY. (**B**–**E**) msZY. v: vacuole, n: nucleus, t: tonoplast, cw: cell wall, c: cytoplasmic, m: mitochondria, og: osmophilic granules. Scale bars in (**A**) = 5 μm, in (**C**,**D**) = 3 μm, and in (**B**,**E**) = 1 μm.

We observed the ultrastructure of young flower buds of msZY and ZY by transmission electron microscopy (TEM). In ZY, one or a few vacuoles were scattered throughout the cytoplasm, which were clearly delimited by the intact tonoplast, and there were large and clear nuclei in the cells (Figure 6A). In msZY, irregular outlines of vacuoles and enlarged mitochondria (Figure 6B), nuclear envelope disassembly (Figure 6C), and cytoplasmic shrinkage and condensation (Figure 6D) were observed. These are typical characteristics of plant PCD. We also found obvious osmophilic granules in some vacuoles of msZY (Figure 6E). The results of the ultrastructure and transcriptome analyses revealed that active PCD occurred in young flower buds of msZY.

### 2.8. Differentially Expressed Genes Related to the Heat Shock Protein Family

HSPs are major proteins that maintain cell homeostasis and function directly in plant development and stress resistance. In our study, a total of 12 *Hsps* were identified as DEGs, and all of them were significantly downregulated in msZY flower buds (Table S3).

## 3. Discussion

The transcriptome is a collection of all RNAs expressed by specific cells, tissues, and organs over a period of time. The representativeness of a sampling is therefore a matter of concern in transcriptome research. In this study, we focus on the genes and regulatory pathways involved in anther abortion of male sterile lines. Cytological observation showed that the abortion of msZY occurred in the early stages of anther development, before sporogenous cells formation (Figure 1A,B). Because the proportion of anthers in flower buds is very large (Figure 1) in the early stage of anther development, we used flower buds in this stage instead of anthers for subsequent transcriptome sequencing. If the anther-related tissue can be removed under the microscope by laser capture microdissection and then be sequenced, it will undoubtedly make the target of DEGs clearer.

Mitochondrial energy deficiency causes male sterility in some CMS systems [2]. The five complexes of the mitochondrial respiratory chain have different effects on ATP production, and energy metabolism abnormalities associated with CMS focus on the complex V [12]. The subunits of a (atp1, atp6), b (atp4), c (atp9), and A6L (atp8) are encoded by the mitochondrial genome in tobacco [58]. In the present study, four of these five mitochondrial encoded genes were downregulated in msZY, and there was lower ATP content in young flower buds of msZY than in ZY (Figure 4). Abnormal energy metabolism or DEGs enriched in energy metabolism were observed in *pol* CMS [7], *SaNa-1A* CMS [59], and *hau* CMS [15]. Decreased ATP production was considered the reason for pollen abortion in transgenic tobacco [27] and rice [60]. Mitochondrial energy metabolism is mitochondria utilizing oxygen to produce ATP via OXPHOS, so mitochondrial energy metabolism is importantly linked to hypoxia [61]. While redox status is an important determinant of germ cell development in pre-sporogenous stage [62]—for example, hypoxic around the tassel, triggering archesporial cells formation and manipulating redox status—any lobe cell can become an archesporial cell in maize. We considered that low ATP content due to the lack of F1F0-ATPase subunit transcripts might reflect the status of pre-sporogenous developmental stage in msZY flower buds.

In other CMS plants, the sterilizing factors of CMS work by interacting directly or indirectly with OXPHOS complexes, which results in both low ATP production and ROS accumulation. ROS accumulation was more likely to be the cause of CMS [9,10]. The abortion mechanism of plant CMS is a complex process, involving a single factor or multiple factors working together in a plant CMS mechanism, or combined factors that are part of the same mechanisms underlying CMS.

The ER is an essential organelle for correct folding of proteins. Newly synthesized peptides are correctly folded with the help of the ER chaperone. When misfolded proteins increase, the UPR orchestrates restoration of ER homeostasis by enhancing gene expression. In this cell-signaling system, BiP acts as an ER stress sensor in the activation of UPR by binding to IRE1 to repress UPR signaling [63]. Our results showed that the expression level of *BiP* in msZY young flower buds was only 40% of ZY (Table 1). BiP is the most abundant chaperone in the ER lumen that prevents protein aggregation and assists in correct protein folding. Reduction of BiP level was deleterious to the cell viability of tobacco plants [64]. Overexpression of BiP in tobacco [64] and in *Glycine max* [65] alleviated ER stress and osmotic stress-induced PCD.BiPis a negative modulator of PCD. The expression level of *bZIP60* in msZY young flower buds was only 30% of ZY (Table 1). IRE1-bZIP60 is the main branch of the UPR, In *Arabidopsis*, the knockout of key genes *IRE1a* and *IRE1b* in the UPR impairs UPR and enhances PCD [66,67].

The downregulation of *BiP*, *bZIP60*, and other genes in the UPR pathway and ERAD pathway leads to ER imbalance, and PCD is triggered. In the present study, we found that genes *ERD15*, *NAC81*, *NRP-A/B*, and *VPE* in the PCD pathway were upregulated in msZY (Table 1), suggesting that there were active PCD processes in certain cells of young msZY flower buds. The *BI-I* expression profile also strongly supports the above view. Aberrant PCD is an abortion mechanism of some CMS systems. Overproduction of ROS is the major retrograde signal that triggers plant PCD. In CMS –WA (wild abortive) rice, the CMS gene *WA352* interacts with the nuclear-encoded protein COX11 to inhibit the function of COX11 in scavenging ROS, resulting in PCD of the tapetum and then microspore abortion [9]. Excessive ROS production and PCD abnormalities were also observed in the four-stage and early-nuclear stages of HL–CMS rice anthers [68]. Du et al. [59] observed over-accumulation of ROS and signals of PCD in tetrads of SaNa-1A of *B. napus*.

The ER stress pathway is another major signal triggering plant PCD. Treatment of soybean cells with cyclopiazonic acid, a specific elicitor of plant ER stress, results in ER stress, followed by the induction of a cell death program [69]. Treatment of *Arabidopsis* seedlings with tunicamycin (TM), an inducer of ER stress, results in the strong inhibition of root growth accompanied by typical hallmarks of PCD [45]: drought, heat, and biotic stress-induced plant ER stress, as well as PCD in *Arabidopsis* [70]. In addition, most Hsps were downregulated in msZY (Supplementary Table S3). *HSPs* are reported to negatively regulate PCD [50,71]. The downregulation of Hsps was thus consistent with active PCD in young flower buds of msZY.

## 4. Materials and Methods

### 4.1. Plant Materials

The tobacco *sua*-CMS line msZY (an isonuclear alloplasmic line of ZY) and the male fertile control ZY were planted in the experimental field of the Tobacco Research Institute in Qingdao, Shandong province in China.

### 4.2. Microscopic Observation of Early Anther Development

Flower buds <2 mm and 2–3 mm in length were picked and fixed in FAA solution after vacuum treatment. Fixed flower buds were dehydrated by ethanol in a step-graded manner and embedded in wax. Blocks were cut 5 μm thick, and sections were examined under a stereomicroscope (SZX12, Olympus, Tokyo, Japan). Then, sections were stained with hematoxylin and observed using a microscope (Nikon 80i, Nikon, Tokyo, Japan) equipped with a CCD.

### 4.3. RNA-Seq

Flower buds <2 mm in length were collected from msZY and ZY. Each plant material had three biological replicates. Total RNA was isolated using Plant RNA Extraction Kit (TaKaRa, Dalian, China) according to the standard protocol. A NEBNextUltraTM RNA Library Prep Kit (NEB, United States) was used to generate sequencing libraries, which were sequenced using a paired-end read protocol, with 100 bp of data collected per run on an Illumina Hiseq 2000. The cDNA library preparation and Illumina sequencing were conducted by the Novogene Company (Beijing, China). The quality of cDNA libraries was assessed on an Agilent Bioanalyzer 2100 and an ABI StepOnePlus Real-Time PCR system. The quality of the sequencing data was scrutinized in terms of total raw reads, total clean reads, Q20 percentage, and GC percentage.

### 4.4. Sequence Assembly and Differentially Expressed Gene (DEG) Analysis

Before assembly, low-quality sequences (reads with quality values ≤20) were filtered to obtain high-quality clean data (clean reads). Reference genome and gene model annotation files were downloaded from NCBI (https://www.ncbi.nlm.nih.gov/genome/425). The paired-end clean reads were aligned to the reference genome of tobacco using HISAT2 (v2.0.5) [72].

HTSeq was used to estimate gene expression levels of each sample. The DEGs were identified using the DESeq2 R package (v1.16.1, Genome Biology Unit, European Molecular Biology Laboratory, Meyerhofstrasse, Heidelberg, Germany) [73]. The resulting *p*-values were adjusted using Benjamini and Hochberg’s approach for controlling the false discovery rate. Genes with an adjusted *p*-value < 0.001 found by DESeq2 were assigned as differentially expressed. The relative expression of unigenes were calculated using the fragments per kb per million reads (FPKM) method.

Gene ontology (GO) functional and Kyoto Encyclopedia of Genes and Genomes (KEGG) pathway analyses were carried out on the screened DEGs. GO enrichment analysis of the DEGs was implemented using the Blast2GO platform [74], in which gene length bias was corrected. GO terms with corrected *p*-values < 0.05 were considered significantly enriched by DEGs. KEGG analysis used clustering from the Profiler R package to test the statistical enrichment of DEGs in KEGG pathways (corrected *p*-value < 0.05).

### 4.5. qRT-PCR Analysis

To validate the transcriptome data and characterize genes that were differentially expressed between msZY and ZY, the relative expression levels of 16 genes were checked in the young flower buds (<2 mm) of msZY and ZY by qRT-PCR. The cDNAs were synthesized using HiScript Ш RT Super MIX for qPCR (+gDNA wiper) (Vazyme, Nanjing, China), according to the manufacturer’s protocol. Primers (Supplementary Table S1) were designed based on reference unigene sequences with an online primer design tool (http://www.ncbi.nlm.nih.gov/tools/primer-blast/). The tobacco ACTIN gene was used as the internal control for normalization. qRT-PCR was performed using the ChamQTMSYBR Color qPCR Master Mix (Vazyme) in a Light-Cycler96 SW (Roche, Switzerland) with three technical replicates. Analysis of the relative gene expression data was conducted using the 2^−ΔΔCt^ method [75].

### 4.6. Quantification of ATP

The ATP content of young flower buds of msZY and ZY was quantified using acolorimetric-based ATP Assay Kit (Comin Biotech, Suzhou, China). The fresh young flower buds were ground immediately on ice with acid extract from the ATP Assay Kit. Centrifugation was performed at 8000× *g* for 10 min at 4 °C to extract ATP. The supernatant was transferred to a new tube, the same volume of alkaline extract was added, and the samples was mixed and centrifuged at 8000× *g* for 10 min at 4 °C. The supernatant was placed in a new tube on ice for the ATP assay. The absorbance at 700 nm was proportional to the amount of ATP. The results are expressed as μM ATP/mg fresh weight based on a standard curve of known ATP amounts. All of the experiments were repeated in triplicate.

### 4.7. Transmission Electron Microscopy

Young flower buds were fixed and saved in 2.5% glutaraldehyde after being vacuum treated. The samples were then rinsed three times with 0.1 M phosphate buffer (pH 7.4) for 15 min each time. After incubating in PBS buffer (1% osmic acid, 0.1M phosphate buffer, pH 7.4) at room temperature for 5 h, the samples were rinsed three times with 0.1 M phosphate buffer (pH 7.4) and dehydrated through a step-graded procedure with ethanol and embedded in resin. Blocks were sectioned using a Leica Ultracut (LeicaUC7, Leica, Vienna, Austria), and sections (60–80 nm) were double-stained with uranium lead citrate, and dried overnight at room temperature. The sections were observed using a transmission electron microscope (HITACHI HT7700).

## 5. Conclusions

This study first determined the abortion stage and the size of flower buds at abortion, and then compared the transcriptomes of young flower buds at the abortion stage in *sua*-CMS line msZY and male fertile control ZY. The cytological analysis indicated that the abortion of *sua*-CMS occurred before the differentiation of sporogenous cells. A total of 462 DEGs, which were enriched in protein processing in the ER, oxidative phosphorylation, photosynthesis, and circadian rhythm-plant were identified between msZY and ZY. Genes involved in ER stress pathway, HSP family, F1F0-ATPase, and the differentiation of stamens were downregulated, and genes in the PCD pathway were upregulated in msZY. Ultrastructural and physiological analyses indicted active vacuole PCD and low ATP content in young msZY flower buds. This result suggests that the sterility gene in *sua*-CMS mitochondrion might suppress expression of the ER stress pathway and early anther development in the nucleus, resulting in ER stress-induced PCD. PCD and the deficiency of ATP synthesis are essential in the abortion of *sua*-CMS. This study helps to understand the mechanism of anther abortion in early anther development and facilitate the breeding of tobacco hybrids.

## Figures and Tables

**Table 1:** DEGs related to endoplasmic reticulum(ER) stress, programmed cell death(PCD), anther development, and F1F0-ATPase.

Pathway of Function	Gene	Gene ID	msZY-FPKM	ZY-FPKM	Fold Change (msZY/ZY)	*p*-Value
ER stress	*BiP*	LOC107771425	27.5	64.4	0.4	1.2 × 10^−7^
	*bZIP60*	LOC107761889	14.2	49.1	0.3	0.00014685
	*CNX*	LOC107772248	62.7	81.4	0.8	0.041817
	*CRT*	LOC107814655	156.2	197.9	0.8	2.60 × 10^−81^
	*HRD1*	LOC107832607	1.1	1.4	0.8	1.25 × 10^−76^
	*HRD1*	LOC107781322	7.9	9.4	0.8	8.35 × 10^−75^
	*HRD3A*	LOC107799383	6.5	8.7	0.7	1.03 × 10^−74^
PCD	*ERD15*	LOC107831960	58.5	12.4	4.7	0.00000922
	*NAC81*	LOC107784516	0.6	0.4	1.6	0.0058003
	*NRP-A/B*	LOC107799405	200.1	88.0	2.3	0.0 20581
	*NRP-A/B*	LOC107786583	150.6	59.7	2.5	0.04181
	*VPE*	LOC107807349	5.5	3.0	1.9	0.0016425
	*BI-1*	LOC107803860	0.6	1.1	0.5	0.046784
Anther development	*SPL/NZZ*	LOC107789918	0.21	5.50	0.04	1.25× 10^−7^
	*PI*	LOC107803851	25.48	51.21	0.50	0.0 20896
	*AG*	LOC107761813	15.13	23.30	0.65	0.0022628
F_1_F_0_-ATPase	*atp9*	-	276.2	432.9	0.6	0.0012869
	*ORFB(atp8)*	-	73.7	122.0	0.6	5.92 × 10^−119^
	*atp6*	*-*	692.0	1104.6	0.6	2.35 × 10^−260^
	*atp4*	*-*	146.9	212.0	0.7	1.75 × 10^−16^

## Supplementary Materials

Supplementary materials can be found at https://www.mdpi.com/1422-0067/21/7/2445/s1.

## Abbreviations

ER	endoplasmic reticulum
KEGG	Kyoto Encyclopedia of Genes and Genomes
PCD	programmed cell death
CMS	cytoplasmic male sterility
TFs	transcription factors
AP3	APETALA3
PI	PISTILLATA
AG	AGAMOUS
NZZ/SPL	NOZZLE/SPOROCYTELESS
OXPHOS	oxidative phosphorylation
ROS	reactive oxygen species
TCA cycle	tricarboxylic acid cycle
ERQC	ER quality control
UPR	unfolded protein response
BiP	binding protein
CNX	calnexin
CRT	calreticulin
PDI	protein disulfide isomerase
IRE1	inositol-requiring enzyme 1
ERAD	ER-assisted degradation
NRP	N-rich protein
NAC	NAM, ATAF1, CUC2
VPE	vacuolar processing enzyme
BI-1	Bax inhibitor 1
HSPs	heat-shock proteins
DEGs	differentially expressed genes
TEM	transmission electron microscopy
TM	tunicamycin
FPKM	fragments per kb per million reads
